# Erionite-Na upon heating: dehydration dynamics and exchangeable cations mobility

**DOI:** 10.1038/srep22786

**Published:** 2016-03-07

**Authors:** Paolo Ballirano, Alessandro Pacella

**Affiliations:** 1 Department of Earth Sciences, Sapienza University of Rome, Piazzale Aldo Moro 5, I-00185, Rome, Italy; 2 Rectorial Laboratory Fibres and Inorganic Particulate, Sapienza University of Rome, Piazzale Aldo Moro 5, I-00185, Rome, Italy

## Abstract

Erionite is a fibrous zeolite significantly more tumorigenic than crocidolite asbestos upon inhalation. In recent years, several papers have been published aimed at characterizing from the crystal-chemical point of view erionite fibres. As their toxicity has been ascribed to Fe acquired within the human body, studies aimed at characterizing the iron topochemistry have also been published, suggesting a possible important role played by the ionic exchange properties and cations mobility of this zeolite on developing carcinogenicity. Here we report the analysis results of the thermal behaviour of erionite-Na, which has been found to deviate significantly from that of erionite-K. This result is in contrast with the current scientific view that differences in weighted ionic potential, Si/Al ratio and size of exchangeable cations result in significantly different thermal behaviours, all those parameters being nearly identical or very similar in both species. The different mobility of the extraframework cations observed in erionite samples with dissimilar chemistry is of particular interest within the frame of the hypothesis that their biological activity could depend, apart from surface interactions, also on bulk effects.

Erionite is a relatively uncommon zeolite, characterized by fibrous morphology^1^,^2^,^3^, which has been proved, by *in vivo* studies, to be significantly more tumorigenic than crocidolite upon inhalation^4^. Accordingly, the International Agency for Research on Cancer classified it as a Group 1 known Human-Carcinogen^5^,^6^. Erionite pertains to the so-called ABC-6 family^7^, which is described on the basis of the stacking along the c-axis, following an ABC scheme, of layers of six-membered rings made of (Si,Al)O_4_ tetrahedra. Among the various members, erionite possesses a six-layer repetition. In theory, permitting the occurrence of both single- and double-rings, 10 different stacking sequences are allowed for a period of six-layers^8^,^9^. To date, only four of them have been found in minerals: AABAAC, erionite^10^, AABBCC, chabazite^11^, ABBACC, bellbergite^12^, and ABABAC, liottite^13^. Erionite has a general formula K_2_(Na,Ca_0.5_)_8_[Al_10_Si_26_O_72_] • 30H_2_O but it experiences a relevant chemical variability that had led to the recognition of three different species, erionite-K, erionite-Na, and erionite-Ca, according to the prevailing extraframework (EF) cation^14^. A relevant chemical variability has been observed also at the same locality (e.g., Rome, Oregon, USA^15^,^16^,^17^). The framework of erionite consists of two cancrinite (ε) cages, two double-6 rings (D6R), and two erionite cages per unit cell. EF cations are hosted by both cancrinite and erionite cages. The cancrinite cage contains a K ion (site K1) at its centre, whereas three EF cation sites, named Ca1, Ca2, and Ca3, have been identified along the axis of the erionite cage, plus an additional K2 site. This site is located at the centre of the boat-shaped 8-member rings (8MR), which forms the walls of the erionite cage, and it hosts extra-K ions^15^. An interesting, albeit overlooked, aspect among the various physical-chemical properties of erionite is its thermal behaviour. To date, only the dehydration dynamics and the thermal stability of erionite-K have been investigated^18^. The main results obtained were:

- the structural breakdown starts at 1113 K and is completed at 1193 K;
- the dependence of cell parameters and volume on temperature is complex;
- the Ca1, Ca2 and K1 sites deplete as temperature is raised as a result of an EF migration towards K2;
- the reduction of site scattering (*s.s.*) at K1 has been attributed to the so-called “internal ion exchange mechanism”^19^;
- the full depletion of the water molecule sites is attained in the 413–573 K thermal range although OW7 shows relevant *s.s*. up to the breakdown temperature;
- the persistence of *s.s*. at OW7 has been attributed to the migration of EF cations at that site.

Here we shall investigate the thermal stability of erionite-Na by *in-situ* High-Temperature X-ray Powder Diffraction (HT-XRPD) in order to compare its behaviour with that of erionite-K. A hand specimen from Rome, Oregon, USA, where both species coexist, was used in the present work testifying the significant chemical variability of this zeolite. This is a crucial point as erionite from Rome has been extensively used in the past for both *in-vivo* and *in-vitro* studies without a detailed crystal chemical characterization. We will demonstrate that the different EF cations content and position within the erionite cavity result in contrasting thermal behaviours. The finding of a diverse thermally-induced mobility path of the EF cations is a result of interest as it has been hypothesized that the biological activity of erionite could depend, apart of surface interactions, also on bulk effects favoured by the cation exchange properties of this zeolite^17^. Such study is framed within a broad research project intended to characterize at the crystal chemical and structural level and to investigate the chemical-physical properties of erionite fibres, the final target being to disclose a possible route of inactivation of their carcinogenicity.

## Results

### Cell parameters and volume dependence on temperature

**Figure 1:**
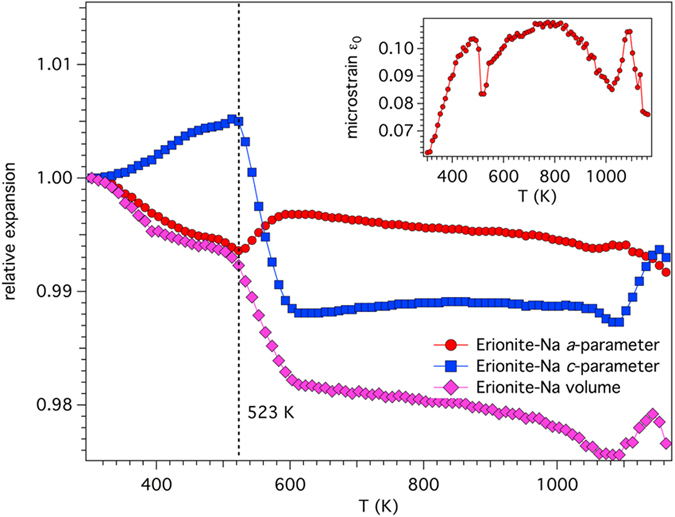
Relative expansion of cell parameters and volume of erionite-Na. Relative expansion of cell parameters (*a*/*a*_303K_ and *c*/*c*_303K_) and volume (*vol*/*vol*_303K_) of erionite-Na. Inset: dependence of e_0_ microstrain on temperature.

Erionite represents one of the several microporous materials showing a negative thermal expansion^18^. The dependence of cell parameters and volume of erionite-Na on temperature are reported in Supplementary Fig. 1 and the dependence of the relative expansion (*a*/*a*_303K_, *c*/*c*_303K_, *vol*/*vol*_303K_) on temperature in Fig. 1. Comparison of present data with those of erionite-K points out to significant differences and in some cases contrasting behaviours. The *a*-parameter of erionite-Na decreases up to 523 K and then increases back, up to 613 K. As temperature is further raised, the *a*-parameter irregularly decreases, with the exception of a minor increase in the 1063 K ≤ T ≤ 1103 K thermal range, up to the temperature of breakdown (T_break_). The collapse starts at 1063 K and is completed at 1173 K. Therefore, erionite-Na has a T_break_ slightly smaller than that of erionite-K but still higher than that reported in a recent comprehensive review on the behaviour of zeolites upon heating^20^. The *c*-parameter increases, similarly to erionite-K, up to 523 K. However, as temperature is raised, it contracts abruptly of ca. 1.7% decreasing from ca. 15.14 Å to ca. 14.88 Å in the relatively small temperature range of 100 K. Passing from 613 K to ca. 1053 K there are only marginal variations before observing the absolute minimum of 14.8716(3) at 1093 K. Finally, the *c*-parameter steadily increases until the breakdown is reached. Despite the substantial differences reported for the evolution of the cell parameters as a function of temperature of erionite-Na and -K that of cell volume is similar. It can be summarized as a contraction, albeit magnified in the case of erionite-Na, up to 1083 K followed by an expansion up to 1143 K, before a final compression until structural disruption. The maximum cell volume contraction with respect to the largest cell volume ΔV_max_ is of ca. 2.5%, a value nearly double than that reported for erionite-K.

The dependence of microstrain ε_0_ (lattice strain, defined as β_i_ = 4ε_0_ tan θ^21^), on temperature has been also evaluated (inset of Fig. 1). Microstrain increases from a value of ca. 0.06 at 303 K to ca. 0.10 at 523 K. In correspondence to the discontinuity observed for both *a*- and *c*-parameter there is a partial release of microstrain, which slowly returns to the value of 0.10. A second partial release is observed in correspondence to the discontinuity detected for both *a*- and *c*-parameter at approximately 1050 K. A further fast increase to 0.10 is followed, in correspondence to the starting of the structural breakdown, by a release of microstrain until the complete structure disruption is attained. This overall behaviour is markedly different with respect to that observed in erionite-K coherently with a different dependence of the cell parameters on temperature^18^.

Despite the low content of admixed chabazite (space group *R* 3*m*) of ca. 3 wt.%, we were able to describe the dependence of the *a*-parameter and the *α*-angle on temperature (Supplementary Fig. 2).

### Structural modifications

**Figure 2:**
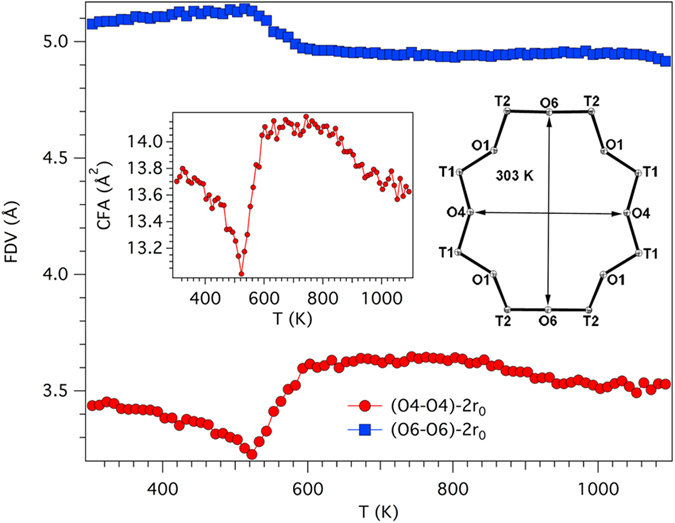
Dependence of the shape of 8MR on temperature. The evolution of the shape of the 8MR as a function of temperature. See text for details of FVA calculation. Inset: dependence of the Crystallographic Free Area (CFA) on temperature. Area calculated approximating the ring shape to an ellipse. The shape of the channel at 303 K is also displayed (ORTEP-3).

**Figure 3:**
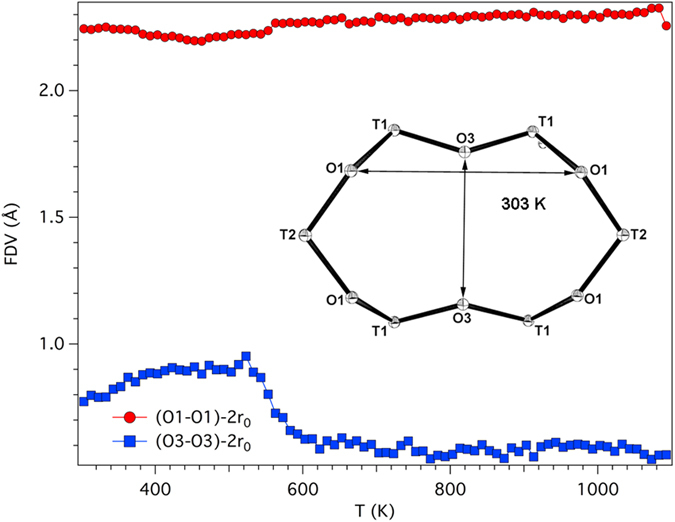
Dependence of the shape of the 6-member ring (6MR) on temperature. The evolution of the shape of the six-membered ring shared between neighbouring cancrinite and erionite cages as a function of temperature. The shape of the ring at 303 K is also displayed (ORTEP-3).

The <T2-O> bond distance regularly decreases as temperature is raised whereas <T1-O> is almost unaffected by temperature (Supplementary Fig. 3). This is a behaviour similar to that of erionite-K. The reported values at 303 K of <T1-O> = 1.632 Å and <T2-O> = 1.654 Å are in good agreement with those reported at RT (<T1-O> = 1.630 Å, <T2-O> = 1.652 Å) for a sample of similar composition^17^. From Jones’ determinative curves^22^, a distribution of 4.36 Al atoms per formula unit (*apfu*) at T1 and 3.87 Al *apfu* at T2 site has been calculated leading to an R value [R = Si/(Si + Al)] of 0.771, which compares favourably with 0.789 from the regression equation of Passaglia *et al.*^23^ and 0.796 from SEM-EDX data. The dependence of the T-O-T angles on temperature is shown in Supplementary Fig. 4. The evolution is different with respect to erionite-K. Only T1-O3-T1, T1-O4-T1, and T2-O6-T2 are characterized by significant modification, with differences between the minimum and maximum angle of ca. 10–15°. Both T1-O3-T1 and T2-O6-T2 increase up to 523 K and subsequently contract to a value smaller than that at 303 K. As in the case of erionite-K, the T2-O6-T2 bridge reaches a value close to 180° during its expansion. T1-O4-T1 has a complicated behaviour as it contracts up to 523 K and subsequently expands up to 603 K. Above that temperature it decreases smoothly by a few degrees. The variation of T-O-T angles affects both shape and dimension of the rings delimiting the walls of the cages. The dependence of the shape of 8MR on temperature is reported in Fig. 2. We obtained the minimum and the maximum Free Diameter Values (FDV) by subtracting 2 times the oxygen radius (taken as 1.35 Å)^24^ from, respectively, the O4-O4 and the O6-O6 distance^18^. Moreover, the dependence of the Crystallographic Free Area (CFA) on temperature (*sensu* ref. 25) is also reported. We calculated the CFA by matching the shape of the ring to an ellipse with minor- and major-axis corresponding to the minimum and maximum FDV, respectively. Owing to the different dependence of T-O-T bridges on temperature in erionite-Na and –K, the evolution of CFA from temperature is also different. Similarly to erionite-K, CFA experiences a strong contraction from ca. 13.8 to 13.0 Å^2^ passing from 303 to 523 K. Differently from erionite-K, CFA expands up to a value of ca. 14.1 Å^2^ in the subsequent 80 K. The area remains constant for approximately 200 K before linearly decreasing to ca. 13.6 Å^2^ at the structural breakdown. We report in Fig. 3 the evolution with temperature of the shape of the 6-member ring (6MR) shared between neighbouring cancrinite and erionite cages. This aperture eventually represents the only possible cation diffusion path between the two cages leading to the so-called “internal ion exchange” mechanism^18^,^19^,^26^. By comparison with the behaviour of erionite-K a relevant difference is observed. In fact, whereas in erionite-K the smaller FDV (O3-O3) regularly increases from ca. 0.8 Å at 303 K to ca. 1.1 Å near the structural breakdown^15^, in erionite-Na the maximum value of ca. 0.9 Å is reached at 523 K before a sudden reduction to ca. 0.6 Å at 623 K. Above this temperature the value remains constant up to the breakdown. This behaviour follows that of the T1-O3-T1 bridge, the T-O bond distances being substantially unchanged as a function of temperature. Similarly to erionite-K the larger FDV (O1-O1) shows minor variation.

**Figure 4:**
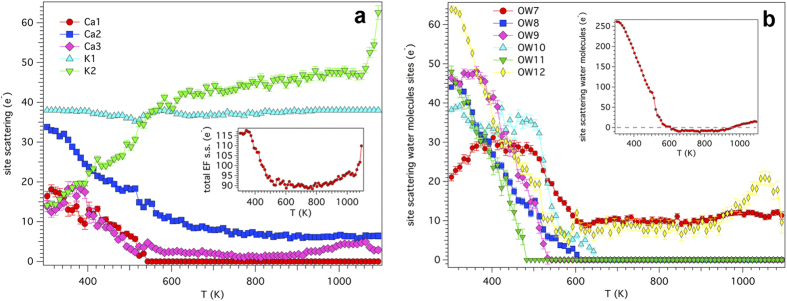
Evolution with temperature of the site scattering of the EF cation sites and of the water molecules sites. (**a**) Evolution with temperature of the site scattering of the Ca1, C2, Ca3, K1, and K2 EF cation sites. Inset: dependence of the total EF *s.s*. on temperature. (**b**) Evolution with temperature of the site scattering of the water molecules sites. Inset: dependence of the total site scattering of water molecules sites on temperature (see text for details on calculation).

**Figure 5:**
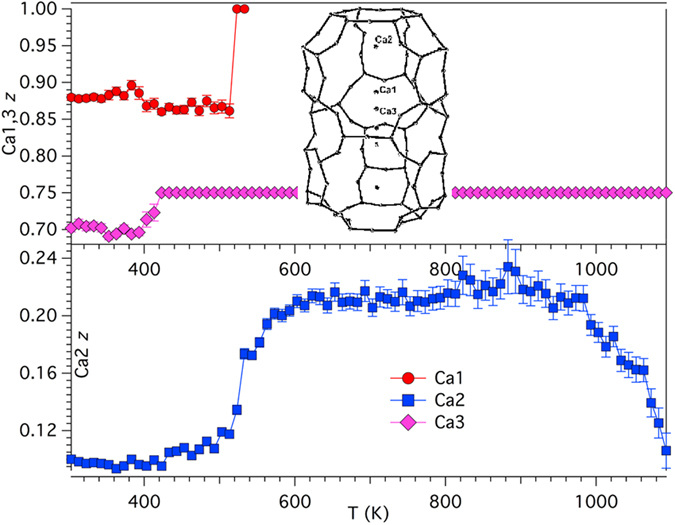
Dependence of the position of EF cations on temperature. Dependence of the *z* coordinate of Ca1, Ca2, and Ca3 on temperature. Inset: location of the three EF cation sites within the erionite cage at 303 K (ORTEP-3).

During the dehydration process EF cations experience both displacement from the position they occupy at 303 K and migration toward different sites. We report the dependence of the site scattering of the EF cations and that of the water molecules sites on temperature in Fig. 4. The dependence of the *z* coordinate (*x* = 1/3, *y* = 2/3) of Ca1, Ca2, and Ca3 sites on temperature is highlighted in Fig. 5. Finally, the evolution with temperature of the *x* coordinate of the OW7 water molecule site is shown in Supplementary Fig. 5. The dependence of the *s.s.* of the EF cation sites on temperature is fairly complex but it may be summarized as a general reduction at Ca1, Ca2, and Ca3 sites counterbalanced by an increase at K2. Moreover, the total EF cation sites *s.s*. tends to decrease as the temperature is raised (see inset of Fig. 4a), at least in the 303–900 K thermal range, suggesting the migration toward low-occupancy EF sites, that were undetected under the present experimental conditions, and/or sites formerly occupied by water molecules before dehydration (see below). More in detail, the reduction of *s.s.* at Ca1 is fairly regular and at temperatures exceeding 533 K the site becomes empty, similarly to erionite-K. The easy mobility of the Mg cations, that are located at Ca1, arises from the fact that at 303 K this site is six-fold coordinated exclusively to water molecules^18^. The depletion of Ca1 correlates with the discontinuity (minimum value) of the CFA of 8MR. The final steps of the depletion are accompanied by a migration of the site from *z* ca. 0.9 to *z* = 1 (special position). Ca2 *s.s*. regularly decreases from ca. 34 e^−^ at 303 K to ca. 7 e^−^ at T_break_. The Ca2 site migrates in the 493–563 K thermal range from *z* ca. 0.1 to *z* ca. 0.2 i.e. approaching the centre of the base of the erionite cavity. The migration positively correlates with the abrupt increase of the CFA of 8MR mainly attained by the increase of the T1-O4-T1 bridge^18^. Differently from erionite-K, Ca2 persists at the same position up to ca. 950 K and subsequently migrates back to the position it occupied at 303 K. Ca3 *s.s.* increases from 14 to 18 e^−^ in the 303–403 K thermal and subsequently regularly decreases to 2 e^−^ at ca. 900 K. In the 900–1063 K thermal range a minor *s.s.* increase to 4 e^−^ is observed followed by a reduction back to ca. 2 e^−^ at T_break_. The *z* coordinate abruptly migrates from ca. 0.7 to *z* = ¾ (special position) in the 403–423 K thermal range. K1 *s.s.* shows minor deviation from the value of 38 e^*−*^ (corresponding to 2 *apfu* K i.e. 1 K atom at the centre of each cancrinite cage) in a restricted thermal range of 483–523 K, which correlates with the maximum expansion of FDV (Fig. 3) of the cancrinite cage. Nevertheless, the maximum value of the (O3-O3)-2r_0_ of 0.9 Å is very short and apparently does not provide clear evidence that the reduction of the site scattering at the K1 site may be explained by invoking a reversible “internal ion exchange” mechanism^18^,^19^,^26^. However, the effect of phonon modes on pore windows breathing could potentially permit such process^27^,^28^. Based on *s.s.* reduction, a maximum 17% substitution of Na for K could be hypothesized.

The total *s.s.* of the EF cation sites at 303 K is of 116(3) e^−^ which is significantly higher than 96(10) e^−^ obtained from chemical data (Supplementary Table 1). The EF cations underestimation is a well-known effect caused by alkali metals migration during SEM-EDX analysis^16^,^17^,^29^. As it has been above mentioned, the total *s.s.* of the EF cation sites fairly regularly decreases to ca. 90 e^−^ except for the final few tens of K before T_break_. In fact, in that thermal range there is a sudden increase related to the strong raise of the *s.s.* at K2 (inset of Fig. 4a). We may hypothesize that such increase is possibly due to the partial unreliability of those refinements, which are potentially affected by the strong reduction of the diffraction intensities occurring near T_break_.

The complete depletion of the various water molecule sites is attained from 483 K (OW11) to 653 K (OW10) with the exception of OW7 and OW12 that show significant *s.s.* (ca. 10 e^−^) up to T_break_ (Fig. 4b). This scheme is similar to that shown by erionite-K^18^ except for the persistence of *s.s.* at OW12 and a general increase of 50–70 K of the temperature of depletion of the various water molecule sites. By hypothesizing that the total *s.s.* of the EF cation sites does not change during the heating process, the dehydration process has been reconstructed. Subtraction of the total *s.s.* of EF cations at 303 K from the sum of the total *s.s.* of the water molecules sites and that of EF cations at each temperature (inset of Fig. 4b) suggests that the complete dehydration is attained in the 550–600 K thermal range. The calculated values show deviations of ± 10 e^−^ from the expected value of 0 e^−^ at complete dehydration, which is a very reasonable result as it is of the same magnitude of the standard deviation of the total *s.s.* of both EF cation and water molecules sites. The observed dehydration kinetics is in excellent agreement with reference TG data^18^,^30^.

The migration of OW7 proceeds regularly from *x* = ca. 0.235 (*y* = 2*x*; *z* = ¾) to *x* = ca. 0.195 i.e. near the walls of the erionite cavity (Supplementary Fig. 5) following a pattern similar to that of erionite-K. This behaviour provides further support to the hypothesis that OW7 represents, in effect, two neighbouring sites occupied by water molecules and EF cations and that the migration is in effect related to the depletion of the site occupied by water molecules. Consistently, the remaining water molecules sites, the exception being OW12, do not show any significant migration providing clear indication that they do not contain EF cations. OW12 coordinates proved to be unstable at temperatures >600 K. This is reasonably due to the ‘dragging’ effect of the neighbouring K2 site (K2-O12 never occurring contact of ca 1 Å) whose *s.s.* significantly increase as temperature is raised. Therefore, its fractional coordinates were kept fixed at the average values (see Methods, Refinement strategy).

## Discussion

We have devised the occurrence of contrasting thermal behaviours in erionite-Na and -K. This result provides clear indication of the dependence of the thermal stability of erionite on its chemical composition. The evolution of the cell parameters with temperature, despite sharing common features with that of erionite-K, results in a maximum volume contraction ΔV_max_ of ca. 2.5%, a value nearly double than that reported for erionite-K. This marked difference is mainly due to a significantly higher compressibility of the *c*-parameter that contracts from ca. 15.14 Å at 523 K to ca. 14.87 Å at 1083 K. This behaviour is apparently in contrast with the higher *c/a* ratio of 1.1392 of erionite-K as compared to 1.1383 of erionite-Na. Complete dehydration is attained in a thermal range (550–600 K) slightly higher than that reported for erionite-K. However, the general mechanism of dehydration is common as it is characterized by significant extraframework cations mobility that occurs via moderate EF sites displacement and intersite rearrangement. EF cations migrate toward the walls of the erionite cage in order to receive a suitable coordination by the oxygen atoms of the framework. The most relevant discontinuity of the process is observed at 523 K and corresponds to the start of a very marked *c*-parameter contraction, which affects the shape of the structural cavities, caused by the complete depletion of the Ca1 site (partially filled by all available Mg) and the almost complete removal of the water molecules. Ca2 migrates toward the centre of the base of the erionite cavity without reaching it, as in the case of erionite-K. As a result, no expansion of the base, required for accommodating Ca2, occurs. Such expansion is attained in erionite-K via widening of T2-O5-T2 and T2-O6-T2 bridges. On the contrary, in erionite-Na T2-O6-T2 contracts, generating a compression along the c-axis of 8MR and, therefore of the whole erionite cage and forcing Ca2 to migrate back to the position occupied at 303 K. Relevant residual electron density has been detected at OW7 and OW12 that has been attributed, as in the case of erionite-K, to EF cations partly migrated from Ca1, Ca2, and Ca3 sites. The reported results contrast with the current scientific view that differences in weighted ionic potential (Z/r)_wt_, Si/Al ratio and size of exchangeable cations should result in significantly contrasting thermal behaviours^20^. On the contrary, the two erionite-K and –Na samples have nearly the same (Z/r)_wt_ and size of exchangeable cations, while showing slightly different Si/Al ratios. This observation points out once more to the difficulty of rationalize the chemical-physical properties of zeolites. The different heat-induced mobility of the EF cations observed in erionite samples with dissimilar chemistry is a result of interest as it may translate in different behaviours whenever fibres are kept in contact with lung fluids^17^. This point is potentially crucial for devising the mechanisms inducing pathogenicity as it has been hypothesized that the biological activity of erionite could depend, apart of surface interactions, also on bulk effects owing to its ionic exchange properties that allow, for example, the segregation of exchanged Fe(II) at a specific crystallographic site^16^, which is a pre-requisite for the formation of reactive oxygen species^31^,^32^. It has been reported the formation of a larger number of foci in human mesothelial cells for North Dakota (ND) samples as compared to those from Turkish villages, the latter characterized by a higher Na^+^/(Na^+^ + Ca^2+^) ratio as compared to those from the USA (ca. 0.5 instead of <0.1)^33^. However, a recent paper has pointed out that, at least at North and South Kildeer Mountain, Dunn County (ND) there is a large incidence of offretite, a related ABC-6 family mineral with AAB sequence, instead of erionite^34^. Moreover, a scrutiny of the ternary diagram reported in Fig. 2 by Carbone *et al.*^33^ suggests that the Mg^2+^/(Na^+^ + Ca^2+^) ratio approaches 1 for almost every analysed fibre, a parameter individuated as discriminating for identify offretite from erionite^23^. Therefore, the results of Carbone *et al.*^33^ indicating the substantial similarity of the biological activity of samples of erionite from different localities should be supplemented by further tests carried out on well-characterized samples of erionite of dissimilar composition as well as of offretite, for which no information about its toxicity has been reported so far.

## Methods

### Sample characterization and data collection

Erionite-Na from Rome, Oregon, USA was used in the present investigation. Micro-chemical analyses, whose results are reported in Supplementary Table 2, were performed using a FEI Quanta 400 SEM equipped with an EDX Genesis EDS system. Operating conditions were: 15 kV accelerating voltage, 11 mm working distance, 0° tilt angle. The final crystal chemical formula was calculated, after renormalization of the chemical analyses hypothesizing a water content of 18.5 wt% (corresponding to *ca*. 30 atoms per formula unit, *apfu*), on the basis of 36 (Si + Al + Fe^3+^) *apfu*. Both coexisting erionite-Na and -K species were analysed. The crystal-chemical formula of erionite-Na is (Na_3.91_K_2.35_Mg_0.71_)[Al_7.36_Si_28.64_O_72.16_] • 29.60H_2_O in close agreement with reference data for samples from the same locality^16^,^17^. The enrichment procedure is detailed in Ballirano and Cametti^17^. The powder was carefully loaded into a 0.7 mm diameter SiO_2_-glass capillary that was kept open at one end. XRPD data were collected *in-situ* in transmission mode using a Bruker AXS D8 Advance diffractometer operating in θ-θ geometry. The instrument is fitted with focusing Göbel mirrors on the incident beam, Soller slits on both incident and (radial) diffracted beams, a PSD VÅNTEC-1 detector, and a heating chamber^35^. A preliminary room temperature (RT) diffraction pattern indicated the occurrence of minor chabazite (ca. 3 wt.%) and nontronite and traces of quartz. Therefore, a mixed Rietveld/Pawley method was adopted to take into account the small contributions of impurities following the procedure described in detail in ref. 14. Peak shape was modelled through FPA (Fundamental Parameters Approach), imposing the following full axial parameters: divergence slit: 0.6 mm, source length 12 mm, sample length and receiving slit length 6.1 mm.

### Rietveld refinement strategy

The starting structural model of erionite consists, a part of the framework atoms, of five EF cation sites Ca1, Ca2, Ca3, K1, and K2; and six water molecules sites (OW7, OW8, OW9, OW10, OW11, and OW12)^36^,^37^. Structural data of chabazite were taken from Ref. 38 allowing for refinement of cell parameters. Structural data of *α*- and β-quartz were taken, at each non-ambient temperature, from the HT investigation of Kihara^39^ were kept fixed. The reference nontronite lattice was taken from Eggleton^40^ i.e. space group *P*3, *a* = 5.26 Å and *c* = 14.92 Å. Following the same procedure adopted in reference refinements of erionite fibres, both the occupancy of all EF cationic and water molecules sites and the isotropic displacement parameters of all sites were refined. Owing to the occurrence of correlations, displacement parameters of the sites were constrained as follow: *B*_T1_ = *B*_T2_; *B*_O1_ = *B*_O2_ = *B*_O4_; *B*_O3_ = *B*_O5_ = *B*_O6_; *B*_K1_ = *B*_K2_; *B*_Ca1_ = *B*_Ca2_ = *B*_Ca3_ = *B*_Ow8_ = *B*_Ow9_ = *B*_Ow10_ = *B*_Ow11_ = *B*_Ow12_ = 2^*^B_Ow7_. An absorption correction for cylindrical samples was performed^41^. The occurrence of preferred orientation was modelled by spherical harmonics (six refinable parameters up to the 8^th^ order) opportunely choosing the number of terms to be used^42^.

A first series of refinements was carried out allowing the optimization of the spherical harmonics terms that were found to be extremely small (as expected for a capillary mount) and constant throughout the analysed thermal range. A structure refined at a given temperature was used as input for the subsequent temperature.

Owing to the fact that the evaluation of the results pointed out to the same observations raised for the refinements of erionite-K, a second series of refinements was performed using the same strategy^18^ *i.e.* 1) keeping fixed, with the exception of OW7, the fractional coordinates of the water molecules to their average value; 2) at each temperature the isotropic displacement parameters of both EF cations and water molecules were kept fixed to the corresponding values obtained from the linear fitting *B*_iso_ = 15.894 + 0.004 T (K) of the first series of refinements; 3) spherical harmonics terms were again optimized). The effect of the restraints applied on the value of the conventional disagreement indices Rp and Rwp was very marginal throughout the investigated thermal range.

The final structural data set was obtained keeping the spherical harmonics terms fixed to the corresponding averaged values (*y*20 = 0.161; *y*40 = −0.012; *y*60 = 0.004; *y*66*p* = 0.009; *y*80 = 0.041; *y*86*p* = −0.019). A magnified view of the full data set is shown, under the form of a pseudo-Guinier plot, in Supplementary Fig. 6. Representative examples of Rietveld plots of the data collected at 523 and 973 K are shown in Supplementary Fig. 7, experimental details of the XRPD data collection and miscellaneous data of the refinements in Supplementary Table 3. Full structural data of erionite at various temperatures have been deposited under the form of CIF files.

## Additional Information

**How to cite this article**: Ballirano, P. and Pacella, A. Erionite-Na upon heating: dehydration dynamics and exchangeable cations mobility. *Sci. Rep.* **6**, 22786; doi: 10.1038/srep22786 (2016).

## Supplementary Material

Supplementary Information

## References

[b1] Sheppard R. A. & Gude A. I. III U.S. Geological Survey Professional Paper 634, (1973).

[b2] Tschernich R. W. Zeolites of the world pp. 156–166 (Geoscience Press, 1992).

[b3] Bargar K. E. & Keith T. E. C. Calcium zeolites in rhyolitic drill cores from Yellowstone National Park in Natural Zeolites 93 (eds. Ming D. W. & Mumpton F. S. ) 69–86 (International Committee on Natural Zeolites, 1995).

[b4] Coffin D. L., Cook P. M. & Creason J. P. Relative mesothelioma induction in rats by mineral fibers: comparison with residual pulmonary mineral fiber number and epidemiology. Inhal. Toxicol. 4, 273–300 (1992).

[b5] IARC IARC Monographs on the evaluation of the carcinogenic risk to humans. Silica and some silicates. Vol. 42, 225–239 (1987). 2824337

[b6] IARC IARC Monographs on the evaluation of the carcinogenic risk to humans. Arsenic, metals, fibres and dusts. Vol. 100, 311–316 (2011). PMC4781271 23189751

[b7] Gottardi G. & Galli E. Natural zeolites pp. 19–21 (Springer-Verlag, 1985).

[b8] Meier W. M. & Groner M. Zeolite structure type EAB: crystal structure and mechanism for the topotactic transformation of the Na, TMA form. J. Solid State Chem. 37, 204–218 (1981).

[b9] Smith J. V. & Bennett J. M. Enumeration of 4-connected 3-dimensional nets and classification of framework silicates: the infinite set of ABC-6 nets; the Archimedean and 6-related nets. Am. Mineral. 66, 777–788 (1981).

[b10] Staples L. W. & Gard J. A. The fibrous zeolite erionite: its occurrence, unit cell and structure. Mineral. Mag. 32, 261–281 (1959).

[b11] Smith J. V., Rinaldi F. & Dent Glasser L. S. Crystal structures with a chabazite framework. II. Hydrated Ca-chabazite at room temperature. Acta Crystallogr. 16, 45–53 (1963).

[b12] Rüdinger B., Tillmanns E. & Hentschel G. Bellbergite – a new mineral with the structure type EAB. Mineral. Petrol. 48, 147–152 (1993).

[b13] Ballirano P., Merlino S., Bonaccorsi E. & Maras A. The crystal structure of liottite, a six-layer member of the cancrinite group. Can. Mineral. 34, 1021–1030 (1996).

[b14] Coombs D. S. *et al.* Recommended nomenclature for zeolite minerals; report of the Subcommittee on Zeolites of the International Mineralogical Association, Commission on New Minerals and Mineral Names. Can. Mineral. 35, 1571–1606 (1997).

[b15] Ballirano P., Andreozzi G. B., Dogan M. & Dogan A. U. Crystal structure and iron topochemistry of erionite-K from Rome, Oregon, USA. Am. Mineral. 94, 1262–1270 (2009).

[b16] Ballirano P. *et al.* Fe(II) segregation at a specific crystallographic site of fibrous erionite: A first step toward the understanding of the mechanisms inducing its carcinogenicity. Micropor. Mesopor. Mat. 211, 49–63 (2015).

[b17] Ballirano P. & Cametti G. Crystal chemical and structural modifications of erionite fibers leached with simulated lung fluids. Am. Mineral. 100, 1003–1012 (2015).

[b18] Ballirano P. & Cametti G. Dehydration dynamics and thermal stability of erionite-K: Experimental evidence of the “internal ionic exchange” mechanism. Micropor. Mesopor. Mat. 163, 160–168 (2012).

[b19] Schlenker J. L., Pluth J. J. & Smith J. V. Dehydrated natural erionite with stacking faults of the offretite type. Acta Crystallogr. B33, 3265–3268 (1977).

[b20] Cruciani G. Zeolites upon heating: factors governing their thermal stability and structural changes. J. Phys. Chem. Solids 67, 1973–1994, (2006).

[b21] Ballirano P. & Sadun C. Thermal behavior of trehalose dihydrate (T_h_) and β-anhydrous trehalose (T_β_) by *in-situ* laboratory parallel-beam X-ray powder diffraction. Struct. Chem. 20, 815–823 (2009).

[b22] Jones J. B. Al-O and Si-O tetrahedral distances in aluminosilicate framework structures. Acta Crystallogr. B24, 355–358 (1968).

[b23] Passaglia E., Artioli G. & Gualtieri A. Crystal chemistry of the zeolites erionite and offretite. Am. Mineral. 83, 577–589 (1998).

[b24] Shannon R. D. Revised effective ionic radii and systematic studies of interatomic distances in halides and chalcogenides. Acta Crystallogr. A32, 751–767 (1976).

[b25] Baerlocher Ch., McCusker L. B. & Olson D. H. Atlas of zeolite framework types 6th revised version pp. 3–9 (Elsevier, 2007).

[b26] Kokotailo G. T. & Lawton S. L. US Patent 3640680, US Patent Office, Washington DC (1972).

[b27] Hammonds K. D., Deng H., Heine V. & Dove M. T. How floppy modes give rise to adsorption sites in zeolites. Phys. Rev. Lett. 78, 3701–3704 (1997).

[b28] Hammonds K. D., Heine V. & Dove M. T. Rigid-unit modes and the quantitative determination of the flexibility possessed by zeolite frameworks. J. Phys. Chem. B102, 1759–1767 (1998).

[b29] Pacella A., Ballirano P. & Cametti G. Quantitative chemical analysis of erionite fibres using micro-analytical SEM-EDX method. Eur. J. Mineral. 10.1127/ejm/2016/0028-2497 (2016).

[b30] Bloise A. *et al.* TG/DSC study of the thermal behaviour of hazardous mineral fibres. J. Therm. Anal. Calorim. 10.1007/s10973-015-4939-8 (2016).

[b31] Schwidder M., Santhosh Kumar M., Klementiev K. V., Pohl M. M., Brückner A. & Grünert W. Selective reduction of NO with Fe-ZSM-5 catalysts of low Fe content I. Relations between active site structure and catalytic performance. J. Catal. 231, 314–330 (2005).

[b32] Zecchina A., Rivallan M., Berlier G., Lamberti C. & Ricchiardi G. Structure and nuclearity of active sites in Fe-zeolites: comparison with iron sites in enzymes and homogeneous catalysts. Phys. Chem. Chem. Phys. 9, 3483–3499 (2007). 17612716 10.1039/b703445h

[b33] Carbone M. *et al.* Erionite exposure in North Dakota and Turkish villages with mesothelioma. PNAS 108, 13618–13623 (2011). 21788493 10.1073/pnas.1105887108 PMC3158231

[b34] Saini-Eidukat B. & Triplett J. W. Erionite and offretite from the Killdeer Mountains, Dunn County, North Dakota, USA Am. Mineral. 99, 8–15 (2014). 10.2138/am.2014.4567 PMC6287636 30542213

[b35] Ballirano P. & Melis E. Thermal behaviour of β-anhydrite CaSO_4_ to 1,263 K. Phys. Chem. Miner. 34, 699–704 (2007).

[b36] Alberti A., Martucci A., Galli E. & Vezzalini G. A reexamination of the crystal structure of erionite. Zeolites 19, 349–352 (1997).

[b37] Cametti G., Pacella A., Mura F., Rossi M. & Ballirano P. New morphological, chemical and structural data of woolly erionite-Na from Durkee, Oregon, USA Am. Mineral. 98, 2155–2163 (2013).

[b38] Yakubovich O. V., Massa W., Gavrilenko P. G. & Pekov I. V. Crystal structure of chabazite K. Crystallogr. Rep. 50, 544–553 (2005).

[b39] Kihara K. An X-ray study of the temperature dependence of the quartz structure. Eur. J. Mineral. 2, 63–77 (1990).

[b40] Eggleton R. A. Nontronite: chemistry and X-ray diffraction. Clay Miner. 12, 181–194 (1977).

[b41] Sabine T. M., Hunter B. A., Sabine W. R. & Ball C. J. Analytical expressions for the transmission factor and peak shift in absorbing cylindrical specimens. J. Appl. Crystallogr. 31, 47–51, (1998).

[b42] Ballirano P. Effects of the choice of different ionisation level for scattering curves and correction for small preferred orientation in Rietveld refinement: the MgAl_2_O_4_ test case. J. Appl. Crystallogr. 36, 1056–1061 (2003).

