# Supplementary material for: Erionite-Na upon heating: dehydration dynamics and exchangeable cations mobility
Source: Sci Rep. 2016 Mar 7;6:22786. doi: 10.1038/srep22786 (PMC4780034; doi:10.1038/srep22786)
Supplement: Supplementary Information [file srep22786-s1.pdf]

Supplementary Information: Erionite-Na upon heating: dehydration dynamics and exchangeable cations mobility.

Paolo Ballirano[1,2,*], Alessandro Pacella[1]

[1]Department of Earth Sciences, Sapienza University of Rome, Piazzale Aldo Moro 5, I-00185, Rome, Italy
[2]Rectorial Laboratory Fibres and Inorganic Particulate, Sapienza University of Rome, Piazzale Aldo Moro 5, I-00185, Rome, Italy
*paolo.ballirano@uniroma1.it

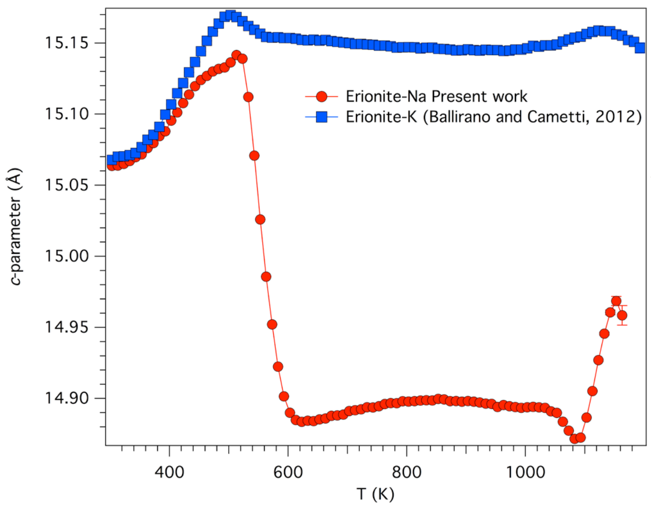

a)

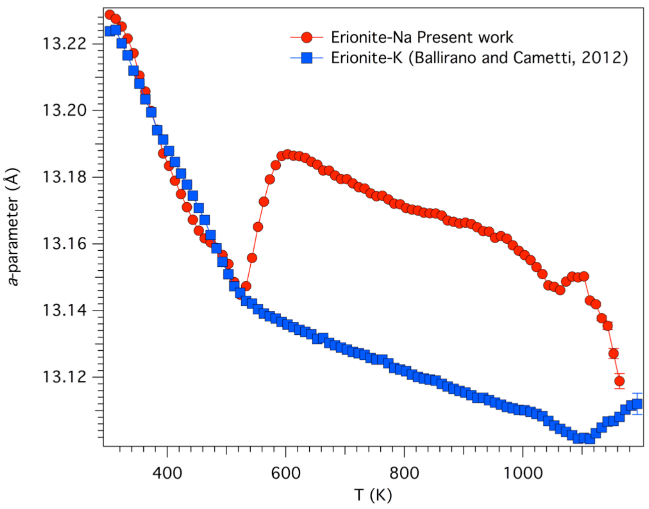

b)

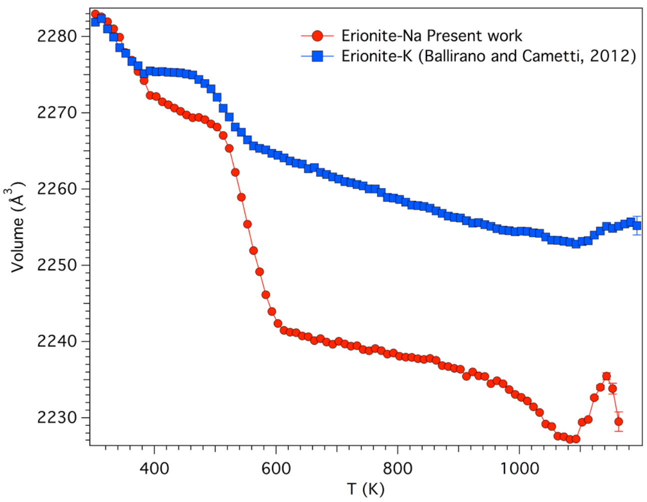

c)

Supplementary Figure 1. **Dependence of cell parameters and volume of erionite-Na on temperature.** Dependence of cell parameters and volume on temperature: **a)** *a*-parameter, **b)** *c*-parameter, **c)** volume. Reference data of erionite-K are reported for comparison[1].

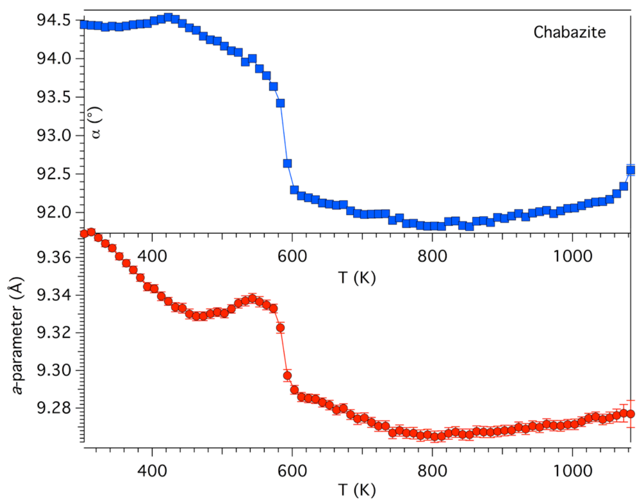

Supplementary Figure 2. **Dependence of the *a*-parameter and α-angle on temperature of chabazite**. Dependence of the *a*-parameter and α-angle on temperature of admixed chabazite. Both *a*-parameter and *α*-angle tend to decrease as temperature is raised. However, the *a*-parameter linearly decreases up to 423 K and subsequently remains substantially unchanged or slightly increases, in the following 150 K. From 573 to 593 K it contracts at a fast rate before realigning to the initial trend. Above 743 K the *a*-parameter is substantially independent from temperature. The *α*-angle is nearly constant up to 423 K and above this temperature it decreases quickly up to 593 K. Above this temperature it suffers from minor modifications. Chabazite has a T_{break} of 1093 K. This thermal behaviour is only fairly consistent with that of siliceous chabazite that has been analysed in the 293-873 K thermal range at five temperatures[2]. In fact those data indicate a regular reduction of volume possibly resulting from the large temperature steps adopted.

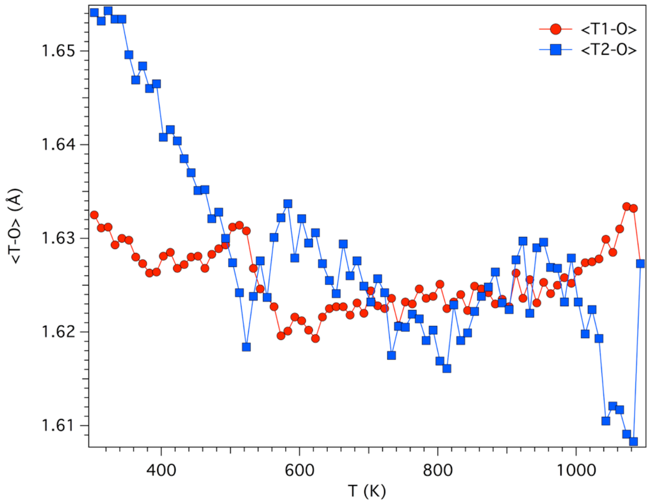

Supplementary Figure 3. **Dependence of <T-O> on temperature.** Dependence of <T1-O> and <T2-O> of erionite-Na on temperature.

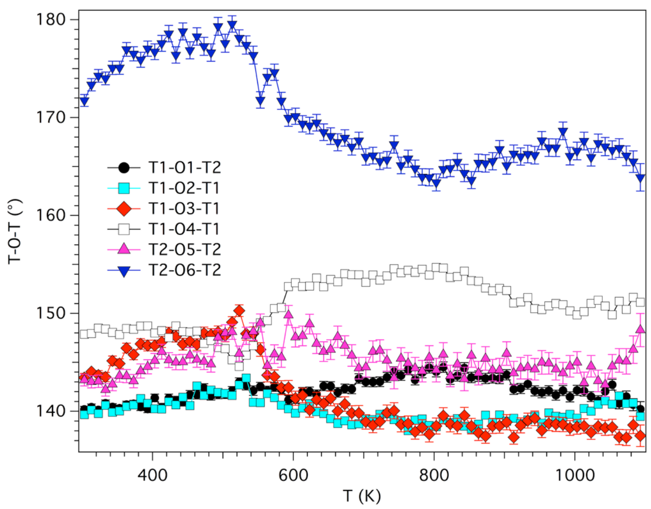

Supplementary Figure 4. Dependence of the T-O-T angles of erionite-Na on temperature. Dependence of the T-O-T angles of erionite-Na on temperature.

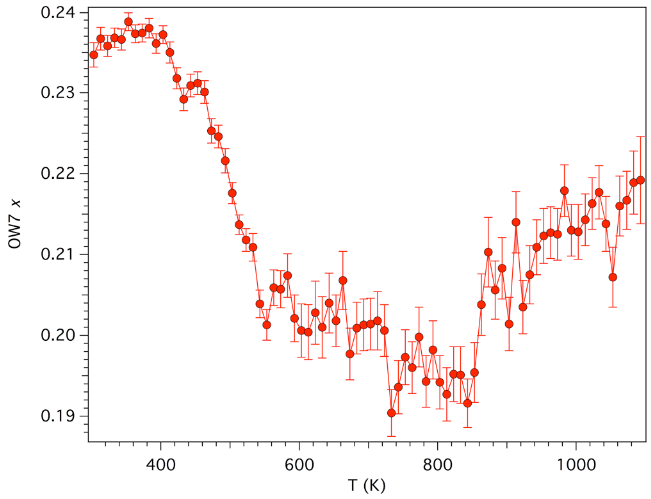

Supplementary Figure 5. Dependence of the *x* coordinate of OW7 site on temperature. Dependence of the *x* fractional coordinate of the OW7 site of erionite-Na on temperature.

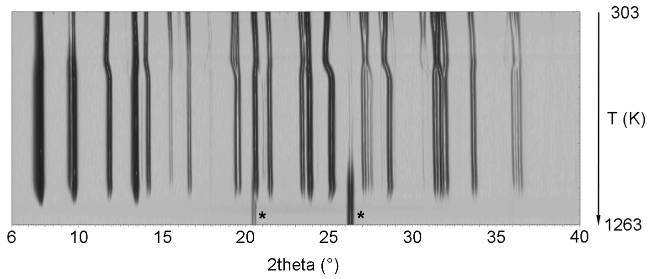

Supplementary Figure 6. Magnified view of the full XRPD data set. Magnified view (6-40° 2θ) of the full data set shown as a pseudo-Guinier plot. * Indicates the position of the relevant reflection of β-quartz occurring because of partial recrystallization of the amorphous material produced by the erionite breakdown.

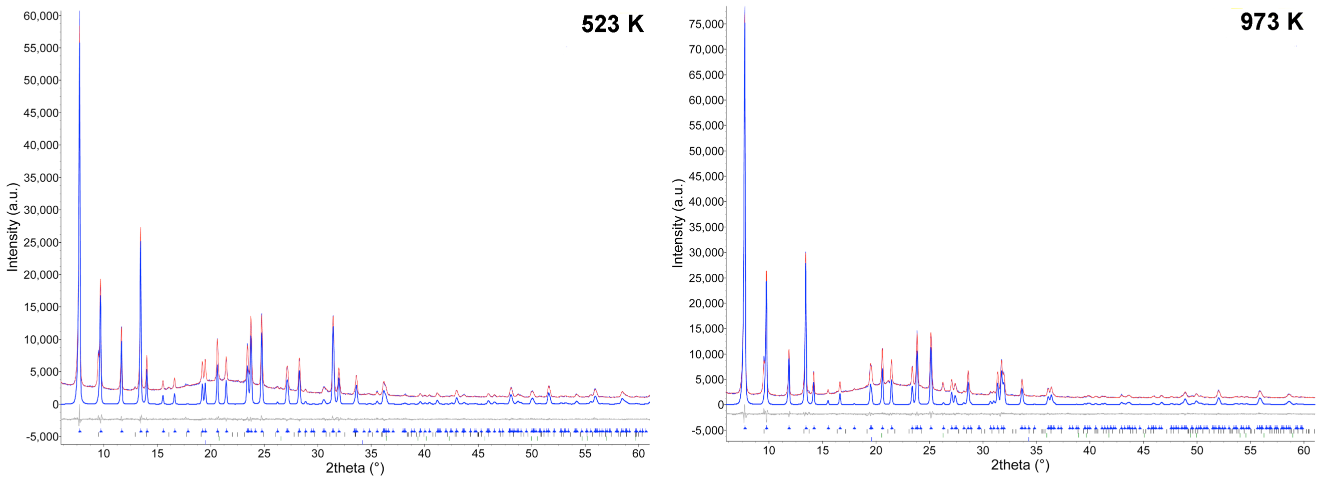

Supplementary Figure 7. **Example of Rietveld plots.** Magnified view (6-60° 2θ) of the Rietveld plots of the data collected at **a**) 523 K and **b**) 973 K. The lower curve represents the difference between observed and calculated plots. Vertical bars indicate to the position of calculated Bragg reflections of, from above to below: erionite, chabazite, quartz, nontronite.

Supplementary Table 1. Cation and water molecule sites scattering at 303 K. Cation and water molecule sites scattering at 303 K from Rietveld refinement and cation site partition from chemical data. The low total EF *s.s.* calculated from chemical data is related to alkali-loss occurring during SEM-EDS analysis[3]. The standard deviation of K content has been arbitrarily assigned to the K2 site, the K1 one being fully occupied. That of Na has been proportionally partitioned between the Ca2 and Ca3 sites.

Site	**Present work at 303 K refinement**	**Partition from chemical analysis**
Ca1 (*s.s.* e^-)	16.4(12)	0.71(8) Mg *apfu* 9(1)
Ca2 (*s.s.* e^-)	33.8(3)	3.1(3) Na *apfu* 34(3)
Ca3 (*s.s.* e^-)	13.9(14)	0.81(7) Na *apfu* 8.9(8)
K1 (*s.s.* e^-)	38.0(0)	2.00 K *apfu* 38.0
K2 (*s.s.* e^-)	14.1(4)	0.4(3) K *apfu* 7(6)
Tot. cations (*s.s.* e^-)	116(3)	96(10)
OW7 (*s.s.* e^-)	21.1(10)	
OW8 (*s.s.* e^-)	44.1(6)	
OW9 (*s.s.* e^-)	46.4(9)	
OW10 (*s.s.* e^-)	38.3(9)	
OW11 (*s.s.* e^-)	48.0(14)	
OW12 (*s.s.* e^-)	63.8(9)	
Tot. water molecules (*s.s.* e^-)	262(6)	

Supplementary Table 2. Chemical characterization of the sample. Chemical analyses, by SEM-EDX, of coexisting erionite-Na and -K occurring in the same hand specimen. R=Si/(Si+Al). Balance error E (%) as defined in Passaglia[4].

Oxides (wt.%)	Erionite-Na	Erionite-K
SiO_2	59.53(61)	59.80(33)
Al_2O_3	12.97(23)	13.18(35)
MgO	0.99(11)	0.88(17)
CaO	-	1.19(17)
Na_2O	4.19(34)	1.56(31)
K_2O	3.82(24)	4.89(35)
H_2O	18.50	18.50
Total	100.00	100.00
Unit cell content (*apfu*)		
Si	28.64(15)	28.57(18)
Al	7.36(15)	7.43(18)
Mg	0.71(8)	0.63(12)
Ca	-	0.61(8)
Na	3.91(34)	1.44(29)
K	2.35(16)	2.98(22)
O	72.16(19)	71.73(23)
H_2O	29.60(37)	29.54(28)
E (%)	-4.1	8.0
R	0.796	0.794

Supplementary Table 3. Crystal data, experimental set-up, data collection, and statistical indicators of the Rietveld refinements. Conventional disagreement indices as defined in Young[5].

Space group	$P6_3/mmc$
Cell parameters at 303 K (Å)	a = 13.22879(14)<br>c = 15.06356(17)
Instrument	Bruker-AXS D8Advance
X-ray tube	Cu operating at 40 kV and 40 mA
Incident beam optics	60 mm multilayer (Göbel) focussing X-ray mirror
Sample mount	Rotating capillary (60 r/min)
Soller slits	2: 2.3° incident beam; radial diffracted beam
Divergence slit	0.6 mm
Detector	PSD VÅNTEC-1
Data range (°2θ)	6-141
Step size (°2θ)	0.022
Counting time (s)	1.5
Thermal range (K)	303-990
Temperature step (K)	10
wRp (%)	2.75-3.59
Rp (%)	2.11-2.76
R_{Bragg} (%)	0.44-0.83

References

1. Ballirano, P. & Cametti, G. Dehydration dynamics and thermal stability of erionite-K: Experimental evidence of the “internal ionic exchange” mechanism. *Micropor. Mesopor. Mat.* **163**, 160-168 (2012).
2. Woodcock, D.A. & Lightfoot, P. Negative thermal expansion in the siliceous zeolites and ITQ-4. A neutron powder diffraction study. *Chem. Mater.* **11**, 2508-2514 (1999).
3. Pacella, A., Ballirano, P. & Cametti, G. Quantitative chemical analysis of erionite fibres using micro-analytical SEM-EDX method. *Eur. J. Mineral.* **DOI: 10.1127/ejm/2016/0028-2497** (2016).
4. Passaglia, E. The crystal chemistry of chabazites. *Am. Mineral.* **55**, 1278-1301 (1970).
5. Young, R.A. Introduction to the Rietveld method in *The Rietveld method* (ed. Young, R.A.) 1-38 (Oxford University Press, 1993).
